# Multiple sources of β2*-nicotinic acetylcholine receptor binding are differentially affected during tobacco smoking abstinence as revealed by Independent Component Analysis of [^18^F]Flubatine PET images

**DOI:** 10.1038/s41386-025-02311-z

**Published:** 2026-01-20

**Authors:** Nakul R. Raval, Katina C. Calakos, Ming-Qiang Zheng, Anita Huttner, Irina Esterlis, Henry Huang, Vince D. Calhoun, Marina R. Picciotto, Kelly P. Cosgrove, Ansel T. Hillmer

**Affiliations:** 1 Department of Radiology and Biomedical Imaging, Yale University School of Medicine, New Haven, CT USA; 2 Yale PET Center, Department of Radiology and Biomedical Imaging, Yale University School of Medicine, New Haven, CT USA; 3 Department of Psychiatry, Yale University School of Medicine, New Haven, CT USA; 4 Department of Pathology, Yale University School of Medicine, New Haven, CT USA; 5 Tri-institutional Center for Translational Research in Neuroimaging and Data Science (TReNDS), Georgia State, Georgia Tech, Emory, Atlanta, GA USA; 6 Department of Radiology, University of Michigan, Ann Arbor, MI USA

**Keywords:** Ion channels in the nervous system, Addiction

## Abstract

Tobacco smoking, a major cause of preventable mortality, upregulates β2 subunit-containing nicotinic acetylcholine receptors (β2*-nAChR) in most brain regions. Although the α4 subunit most frequently co-assembles with β2, other subunits likely assemble with β2 and contribute to distinct aspects of nicotine-mediated behaviors, but these factors are poorly understood in people. This work performed independent component analysis (ICA) of [^18^F]Flubatine positron emission tomography (PET) image data to identify maximally independent sources of specific binding to β2*-nAChRs. We then compared their magnitudes (loading coefficients) in people who recently stopped smoking cigarettes (abstinent smokers; *n* = 26) and people who never smoked cigarettes (non-smokers; *n* = 20). ICA identified 3 reproducible components: IC1 (36% of variance) in medial thalamus, lateral thalamus, and red nucleus; IC2 (18% of variance) in ventral thalamus, lateral geniculate, and midbrain; IC3 (19% of variance) in cerebellum and optic circuitry in midbrain. Nicotine challenge in an independent sample (*n* = 9) reduced loading coefficients of all components, confirming specific binding to nAChRs. Post-mortem autoradiography of cerebellum showed greatest [^18^F]Flubatine displacement by α3/α6β2*-nAChR blocker (α-Conotoxin MII) but low displacement by α6β2*-nAChR blocker (α-Conotoxin PIA), suggesting that IC3 measures α3β2*-nAChRs specific binding – a novel finding in living people. Loading coefficients of IC1 and IC2 were significantly lower in abstinent smokers compared to non-smokers. IC3 loading coefficients were significantly higher during extended smoking abstinence, and exploratory analyses suggested initial evidence for daily smoking amount correlating with nicotine dependence severity. These results could inform novel treatment development to help people quit smoking.

## Introduction

Tobacco use remains a leading cause of preventable death and disease [1]. More than 34 million people in the USA smoke tobacco or use nicotine-related products, of whom ~80% will die from a smoking related disease. Nicotine is the primary addictive component of tobacco and acts in the brain at nicotinic acetylcholine receptors (nAChRs). nAChRs are ligand-gated ion channels composed of 5 subunits, including nine possible α-subunits (α2-α10) and three possible β-subunits (β2−β4) in vertebrates [2]. Nicotine binds to β2-subunit containing nAChRs, referred to as β2*-nAChR to indicate that multiple subunits combine with β2 subunits that regulate the rewarding and motivating properties of nicotine [3, 4]. Ligand binding at β2*-nAChRs occurs at sites located between neighboring pairs of α and β subunits [2]. Chronic nicotine administration robustly upregulates (i.e., increases the number of) β_2_*-nAChRs in most brain regions across animal models, postmortem human brain, and human imaging [5–10]. Positron Emission Tomography (PET) imaging with radiotracers that target β_2_*-nAChRs such as [^18^F]Flubatine, which has high affinity (<10 nM) and selectivity for β_2_*-nAChR, has been a particularly valuable technique to measure these receptors in living people. Indeed, PET imaging studies revealed key clinical findings showing that the magnitude of β_2_*-nAChR radiotracer availability upregulation is associated with craving, cognitive dysfunction [11], and relapse [5–7], thus playing a critical role in continued tobacco use.

Animal studies show that β_2_*-nAChR assembly is complex and has diverse pharmacology and regional distributions in brain. The α4 subunit most commonly co-assembles with β2 subunits, but relative α4/β2 subunit ratios have different sensitivity to nicotine [12, 13], and other subunits including α3, α5, and α6 can combine with β2 and contribute to different aspects of nicotine-mediated behavior in rodent models [2, 14]. More specifically, the α3 and α5 subunits are part of the α3/α5/β4 nAChR gene cluster with an allelic variation that significantly increases risk of nicotine use disorder [15, 16]. Further preclinical results suggest that α3 may be a target for ameliorating cognitive deficits during smoking abstinence [17, 18]. The α6 subunit can be inhibited to reduce nicotine self-administration [19] and alleviate nicotine withdrawal behaviors [20], suggesting this as a target to help with withdrawal symptoms during abstinence [21, 22]. However, translation of these findings to human studies has been limited.

Recently, we developed the novel application of independent component analysis (ICA) to PET imaging data at the group level that can successfully distinguish pharmacologically specific sources of radiotracer binding for the PET radiotracer [^11^C]PHNO, which has high affinity for both dopamine D_2_ and D_3_ receptors. This enabled separate estimates of D_2_ receptor availability and D_3_ receptor availability for each subject [23, 24], which was confirmed with subtype-selective blocking. Motivated by these findings, the goal of the current study was to apply ICA in an analogous fashion to [^18^F]Flubatine PET data obtained from people who recently stopped smoking tobacco cigarettes (abstinent smokers) and from people who never smoked tobacco cigarettes (non-smokers). Specific binding of components to β2*-nAChRs was investigated using an independent dataset that included nicotine-blocking scans with cigarettes and e-cigarettes, and autoradiography in post-mortem human brain tissue. We then compared component loading coefficients between abstinent smokers and non-smokers. We hypothesized that ICA would identify pharmacologically specific pools of β2*-nAChR binding and that these pools would be differentially altered during tobacco smoking abstinence.

## Material and methods

### Study participants and datasets

Image analyses were performed on previously published, independently collected data sets [11, 25], as summarized in Table 1. These studies were approved by the Yale-New Haven Hospital Radiation Safety Committee and the Yale University Human Investigation Committee.

**Table 1:** Participant demographics.

	Dataset I [11]			Dataset II [25]
	Non-Smokers (*n* = 26)	High-Cotinine Abstinent Smokers (*n* = 8)	Low-Cotinine Abstinent Smokers (*n* = 12)	Abstinent Smokers (*n* = 9)
Male; Female (n)	16 M; 10 F	6 M; 2 F	7 M; 5 F	7 M; 2 F
Scans (n)	26 baselines	8 baselines	12 baselines	14 baselines 3 Tobacco Cigarette blocking 6 nicotine 36 mg/mL blocking 5 nicotine 8 mg/mL blocking
Ages (years)	28 ± 6	37 ± 9	37 ± 12	33 ± 12
Race & Ethnicity	2 A, 9 BNH, 7 H, 7 WNH, 1 O	1 A, 7 BNH	2 BNH, 2 H, 7 WNH, 1 O	1 BNH, 1 H, 7 WNH
BMI (kg/m^2^)	25 ± 3	26 ± 2	29 ± 5	29 ± 4
FTCD/FTND	NA	5 ± 2	5 ± 2	4 ± 2
Cigarettes smoked/day (n)	NA	11 ± 2	17 ± 7	*DATA NOT AVAILABLE*
Years smoked (years)	NA	15 ± 11	16 ± 11	*DATA NOT AVAILABLE*
Self-reported Days abstinent *(days)*	NA	7 ± 1	7 ± 1	5
Injected activity and dose (MBq [µg/kg])	234 ± 67 (0.0011 ± 0.0007)	232 ± 70 (0.0007 ± 0.0003)	270 ± 38 (0.0009 ± 0.0006)	261 ± 44 (0.0008 ± 0.0005)

Data presented as Mean ± Standard Deviation.

Smoking characteristics collected at intake prior to abstinence.

High-Cotinine Abstinent Smokers: Individuals exhibiting urine cotinine levels >500 ng/mL.

Low-Cotinine Abstinent Smokers: Individuals exhibiting urine cotinine levels <500 ng/mL.

*BMI* body mass index.

*FTCD/FTND* Fagerström Test for Cigarette/Nicotine Dependence.

*A* Asian, *BNH* Black Not of Hispanic Origin, *WNH* White Not of Hispanic Origin, *H* Hispanic, *O* Other/Unknown.

In brief, **Dataset I** [11] included single scans from 20 participants who recently stopped smoking tobacco cigarettes (abstinent smokers) and 26 individuals who did not smoke tobacco cigarettes (non-smokers). One non-smoker participant reported previously was excluded for this work due to low injected activity (<75 MBq) resulting in noisy voxel-wise images. Those in the abstinent smoker group self-reported smoking at least 5 cigarettes per day for a minimum of one year, with biochemical verification through carbon monoxide (CO) breath output >11 ppm and urine cotinine concentration >150 ng/mL and agreed to quit smoking for up to 2 weeks. Non-smokers smoked fewer than 100 cigarettes in their lifetime and none in the last year, confirmed with a CO breath output <8 ppm and urine cotinine levels <150 ng/mL. This grouping approach is consistent with standard practice for verifying tobacco smoking abstinence [26]. Eight abstinent smokers exhibited urine cotinine levels exceeding 500 ng/mL on the scan day, indicating a “lapse” in smoking abstinence. Individuals exhibiting urine cotinine levels >500 ng/mL are designated as high-cotinine abstinent smokers, while participants with urine cotinine levels <500 ng/mL were categorized as low-cotinine abstinent smokers.

**Dataset II** [25] included 2 PET scans per person, one at baseline (i.e., pre-nicotine) and a second after nicotine self-administration from 6 experienced electronic-cigarette (EC) users and 3 tobacco cigarette smokers. The inclusion criteria for EC users required daily EC use for at least one month, while cigarette smokers were required to smoke at least 10 cigarettes per day. These criteria were confirmed by CO levels >7 ppm and urine cotinine levels >150 ng/mL. Participants agreed to at least five days abstinence prior to imaging to avoid residual nicotine from competing with [^18^F]Flubatine specific binding at baseline. People who smoke tobacco cigarettes (*n* = 3) underwent one blocking scan each with a regular cigarette, while people who smoke E-Cigarettes (*n* = 6) all participated in a scan with 36 mg/mL of nicotine, while (*n* = 5) participated in additional scan with 8 mg/mL of nicotine (details below). Thus, a total of 28 scan sessions (14 baseline and 14 blocking) were included in this dataset.

### Brain imaging

PET scans were acquired using the High Resolution Research Tomograph (Siemens/CTI). Participants wore a Vicra cap (Vicra, NDI Systems) for motion correction and underwent a 6-min transmission scan for attenuation correction. [^18^F]Flubatine was synthesized with high molar activity [27] and administered via computer-controlled pump (Harvard Apparatus) as a bolus plus constant infusion (B/I) (K_Bol_ = 360 min) [28]. Radiotracer infusion lasted at least 120 min for baseline measurements and up to 210 min for nicotine challenge. For challenge scans, participants smoked either electronic cigarettes (ECs) or tobacco cigarettes after 125 min infusion for up to 5 min (see [25]). Dynamic PET data were acquired from 90–120 min for baseline scans and 180–210 min for nicotine challenge scans. For all PET scans arterial blood samples were acquired after initial [^18^F]Flubatine injection to measure the metabolite-corrected arterial input function as previously described [28].

Participants underwent T1-weighted structural magnetic resonance (MR) scans, acquired with a Siemens 3.0 T scanner equipped with a 64-channel head coil. A sagittal gradient-echo MPRAGE sequence was employed (FOV: 256 × 256 mm², 176 slices at 1 mm thickness, TE = 2.77 ms, TR = 2530 ms, TI = 1100 ms, FA: 7°) to provide high-resolution anatomical maps for PET data coregistration.

### Preprocessing and kinetic modeling

List-mode PET data were binned into 5-min frames and reconstructed with corrections for normalization, attenuation, randoms, scatter, deadtime, and head motion using the MOLAR algorithm [29]. MR scans were nonlinearly transformed into Montreal Neurological Institute (MNI) template space and co-registered with early summed kinetic PET images. Motion correction was performed via a six-parameter mutual information algorithm. PET images were denoised using the highly constrained backprojection (HYPR) algorithm [30]. Parametric volume of distribution (*V*_T_) images were generated with equilibrium analysis including correction for radiotracer clearance [31]. Resulting parametric [^18^F]Flubatine *V*_T_ images in MNI template space were smoothed using a Gaussian kernel with a full width at half maximum (FWHM) of 7 mm.

### Independent component analysis

Independent component analysis (ICA) is a data-driven approach used to identify spatial covariation patterns by extracting maximally independent components and their associated signal sources. ICA was performed on an array of [^18^F]Flubatine *V*_T_ whole-brain images to identify spatial components of [^18^F]Flubatine *V*_T_ uptake that share similar patterns of variance across subjects. Spatial ICA was conducted using the Source-Based Morphometry (SBM) module of the Group ICA of fMRI Toolbox (GroupICAT v.4.0c;http://trendscenter.org/software/gift). The primary input were parametric [^18^F]Flubatine *V*_T_ images, which were vectorized and concatenated to form a 2D matrix. Analyses were restricted to a mask containing voxels with a mean (across-subject) *V*_T_ > 8 (see Supplementary Fig. 1), which corresponds to a *BP*_ND_ of 0.23, based on a *V*_ND_ of 6.5 mL/cm^3^ [25, 32]. The global mean *V*_T_ within the mask (*x̄*) was removed from each scan to facilitate component extraction while preserving original *V*_T_ units (mL/cm^3^) in the identified solution. Data were then reduced with principal component analysis followed by ICA using the InfoMax algorithm [33]. ICA was iterated 40 times using ICASSO to determine the most reliable extraction for the final output [34]. The ICA result provides spatial maps of source intensities for each of *i* components (*y*_*I*_*)* and loading values for each subject *j* for each of *m* components (*A*_i,j_). These values can be used to provide an approximation of each subject’s original *V*_T_ map ($\widetilde{{V}_{{{\rm{T}}}}}$) that is the sum of the source products and the global mean value for each scan:

$$\widetilde{{V}_{{{\rm{T}}}}}{{\rm{j}}}=\left({\sum }_{i=1}^{m}{y}_{i}\times {A}_{i,j}\right)+\bar{{x}_{j}}$$

### Evaluation of [^18^F]Flubatine independent components

#### Model-order selection (non-smokers Dataset I; *n* = 26)

ICA was initially performed on the non-smoker control subjects from Dataset I to determine the optimal model order for analysis. Model orders ranging from two to twelve components were evaluated. Across these model orders, the first three independent components (ICs) were consistently retained and captured the majority of the variance, as additional components extracted explained less than 5% of the variance. Stability was also quantified with ICASSO. For model orders 3-12, each of the three components consistently achieved high cluster quality (I_*q*_ > 0.96), indicating robust reproducibility across resampling and informing model-order selection. Based on this observation, a model order of three was selected for further analysis.

#### Regional characterization of ICs (non-smokers Dataset I; *n* = 26)

To analyze the spatial patterns of the extracted ICs across various brain regions, FreeSurfer segmentation (v7.4.1) was employed to calculate the mean $\widetilde{{V}_{{{\rm{T}}}}}$ within specific brain regions. Three segmentation approaches were utilized: basic cortical and subcortical segmentation [35], thalamic nuclei segmentation [36], and PD25 midbrain segmentation [37]. These segmentations were particularly focused on subcortical structures relevant to β2*-nAChR subtype distribution. For each component, the mean $\widetilde{{V}_{{{\rm{T}}}}}$ was calculated, and regions with $\widetilde{{V}_{{{\rm{T}}}}}$ values greater than 1.5 were classified as high-binding regions.

#### Pharmacological validation (non-smokers Dataset I + Dataset II; *n* = 54)

Dataset II adds baseline and nicotine-blocking scans. Combining it with Dataset I allowed us to test: (1) whether the addition of blocking scans altered the source maps, and (2) whether nicotine challenge reduced loading coefficients, hypothesized to represent β2*-nAChR-specific binding. Source-map stability was assessed with Dice similarity coefficients (DSC), and nicotine effects were quantified using the loading coefficients.

#### Component reproducibility (non-smokers + abstinent smokers Dataset I; *n* = 46)

ICA was repeated in only abstinent smokers from Dataset I (*n* = 20), and matched ICs from non-smokers and abstinent smokers were compared with DSC to evaluate component robustness and reproducibility across independent samples. The groups were then pooled, ICA was rerun, and these source maps and loading coefficients were used in final analyses comparing non-smokers with abstinent people who smoked.

### Post-mortem human autoradiography with [^18^F]Flubatine

Post-mortem human brain autoradiography was conducted to assess region-specific binding and blocking potential of [^18^F]Flubatine to various β2*-nAChRs subtypes. The subtypes and corresponding ligands examined were α4β2* (A85380), α6/α3β2* (α-Conotoxin MII), and α6β2* (α-Conotoxin PIA) (Tocris, Bio-Techne Corporation, Minneapolis, MN, USA).

Brain samples were obtained from neurologically healthy individuals (*n* = 3, details in Supplementary Table 1) through the Alzheimer’s Disease Research Center (ADRC) at Department of Neurology, Yale University School of Medicine. Frozen cerebellum (~4-5 cm³) and thalamus (~1 cm³) samples were dissected and sectioned (20 μm) using a cryostat (Leica CM1800, Leica Biosystems, Buffalo Grove, IL, USA). Sections were mounted on Superfrost Plus™ adhesion microscope slides (Thermo Fisher Scientific, Waltham, MA, USA) and stored at −80°C.

On the day of the experiment, [^18^F]Flubatine was synthesized following the same protocol as for PET imaging. Sections were thawed to room temperature, prewashed twice for 10 min in buffer (50 mM Tris-HCl containing 0.5% bovine serum albumin, pH 7.4), and incubated for 60 min in buffer containing 0.1 nM of [^18^F]Flubatine for total binding. Blocking experiments were conducted with 0.1 nM [^18^F]Flubatine and the following ligands at their respective inhibitory constants (*K*_i_): 0.02 nM A85380, 1.5 nM α-Conotoxin MII, and 5 nM α-Conotoxin PIA. These *K*_i_ values were estimated from studies on non-human primate brains (details in Supplementary Information). Incubation was terminated with three 5-min washes in washing buffer (50 mM Tris-HCl, pH 7.4) and a final rinse in 4°C deionized water. After air-drying, the slides were subjected to autoradiography using phosphor image plates (BAS-MS2025, Science Imaging Scandinavia AB, Nacka, Sweden), with exposure times ranging from 45 to 60 min. The plates were then scanned using an Amersham™ Typhoon™ IP (Cytiva, Uppsala, Sweden) at a resolution of 10 µm. Calibration, quantification, and data analysis were performed using ImageJ software (NIH, Bethesda, MD, USA).

### Statistical analysis

To test the hypothesis that the loading coefficients measure specific binding, using Dataset II, paired t-tests were conducted to compare global mean and loading coefficients for each component (IC1, IC2, IC3) before (baseline) and after the nicotine challenge (block) for each condition (NIC8, NIC36, TOB). Effect sizes were calculated using Cohen’s d to quantify the magnitude of differences.

For Dataset I, group differences in demographic and clinical characteristics between non-smokers, abstinent smokers with high cotinine and abstinent smokers with low cotinine were assessed using non-parametric Kruskal–Wallis tests for continuous variables, as most of the variables were not normally distributed based on Shapiro–Wilk tests. Differences in continuous variables across groups were evaluated using the χ² test.

Analyses comparing the global mean and loading coefficients for IC1, IC2, and IC3 between the groups used separate linear models with group as the main factor. Age was included as a covariate in the models for all linear models, as age significantly differed across subgroups. Post hoc pairwise comparisons between groups were conducted with false discovery rate (FDR) correction for multiple comparisons. Effect sizes were calculated using Cohen’s d to quantify the magnitude of group differences.

Associations between smoking-related characteristics and component loading coefficients were conducted separately in high-cotinine and low-cotinine abstinent smoker groups. Pearson’s correlation was used to assess the relationship between loading coefficients (IC1, IC2, IC3) and three clinical characteristics: cigarettes per day, years of smoking, and Fagerström Test for Nicotine Dependence (FTND) scores. To control for multiple comparisons, *p*-values were corrected for multiple comparisons using the Benjamini-Hochberg FDR method.

Statistical significance was set at *p* < 0.05. Statistical analyses were performed using R 4.5.0 (“How About a Twenty-Six”) and RStudio (RStudio Team, Boston, MA, USA), with data visualization carried out using GraphPad Prism (v. 10.4.2; GraphPad Software, San Diego, CA, United States). All reported data are presented as the mean ± standard deviation.

## Results

### Demographics and characteristics

Table 1 shows a summary of study participant demographics and scanning parameters for data used in this study. For Dataset I, Shapiro–Wilk tests indicated that the residuals for age, injected activity, and injected dose deviated from normality, and therefore non-parametric tests were used. A Kruskal– Wallis test revealed a significant difference in age (*p* = 0.0053) across the three groups (non-smokers, high-cotinine abstinent smokers, and low-cotinine abstinent smokers), while no significant differences were observed for body mass index (BMI), injected activity, or injected dose. Sex distribution did not differ significantly between groups.

### Spatial Patterns of ICA-identified Independent Components Source Maps

**Fig. 1:**
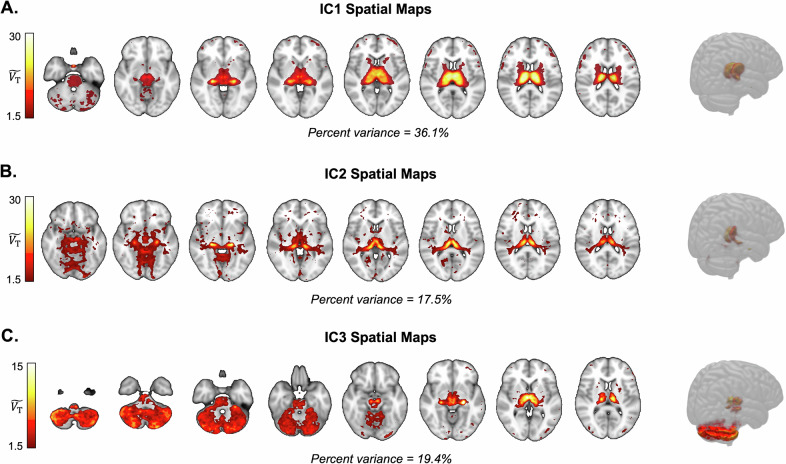
Spatial maps of independent components (IC) derived from ICA across [^18^F]Flubatine PET baseline scans of non-smokers (*n* = 26). IC1 **A**, IC2 **B**, and IC3 **C** are presented in coronal section and 3D view to highlight $\widetilde{{V}_{{{\rm{T}}}}}$ values above 1.5. Note the magnitude of $\widetilde{{V}_{T}}$ scales for different components. Maps are annotated with the corresponding percentage of variance explained for each IC.

ICA was initially performed on the non-smoker group from Dataset I. Spatial patterns of the extracted ICs were subsequently analyzed to delineate areas of high “binding” ($\widetilde{{V}_{{{\rm{T}}}}}$ > 1.5) (Fig. 1 and Supplementary Fig. 2).

#### Independent component 1 (IC1)

IC1 accounted for 36% of the total variance and demonstrated the highest binding primarily within the thalamus. Detailed thalamic segmentation revealed substantial binding across nearly all nuclei, with peak $\widetilde{{V}_{{{\rm{T}}}}}$ values localized in the lateral part of the mediodorsal nucleus and the centrolateral nucleus. Outside the thalamus, regions exhibiting notable binding included the red nucleus, globus pallidus, substantia nigra, and pons.

#### Independent component 2 (IC2)

IC2 explained 18% of the variance and was characterized by a distinct spatial distribution pattern in ventral thalamic nuclei. The highest binding within this component was observed in the pulvinar medial and pulvinar anterior nuclei, followed closely by the medial and lateral geniculate nuclei, as well as the pulvinar inferior nucleus. Additional thalamic nuclei with significant binding included the pulvinar lateral nucleus, lateral posterior nucleus, and central medial nucleus. Binding was also pronounced in extrathalamic regions such as the hippocampus, pons, and parahippocampal cortices.

#### Independent component 3 (IC3)

IC3 represented 19% of the variance and exhibited strong binding predominantly within the cerebellar cortex, cerebellar white matter and thalamus. Within the thalamus, substantial binding was most evident in the central medial nucleus and the lateral part of the mediodorsal nucleus. Other notable thalamic regions included the centromedian nucleus, ventral anterior magnocellular nucleus, ventrolateral posterior nucleus, and ventrolateral anterior nucleus. Midbrain regions, specifically the substantia nigra, demonstrated high binding within this component, with additional substantial binding observed in the red nucleus, subthalamic nucleus, pons, and globus pallidus.

### Component loading coefficients measures of [^18^F]Flubatine specific binding

ICA was subsequently performed on a combined dataset consisting of non-smokers from Dataset I and all scans from Dataset II (total *n* = 54 scans). To verify that inclusion of nicotine blocking scans did not significantly alter the ICA-derived source maps, Dice similarity coefficients (DSC) were computed between source maps generated from non-smokers from Dataset I alone and the pooled dataset. High similarity coefficients (DSC > 0.8 is considered ‘high’ overlap) indicated strong consistency across extracted components (IC1: DSC = 0.90; IC2: DSC = 0.87; IC3: DSC = 0.82).

**Fig. 2:**
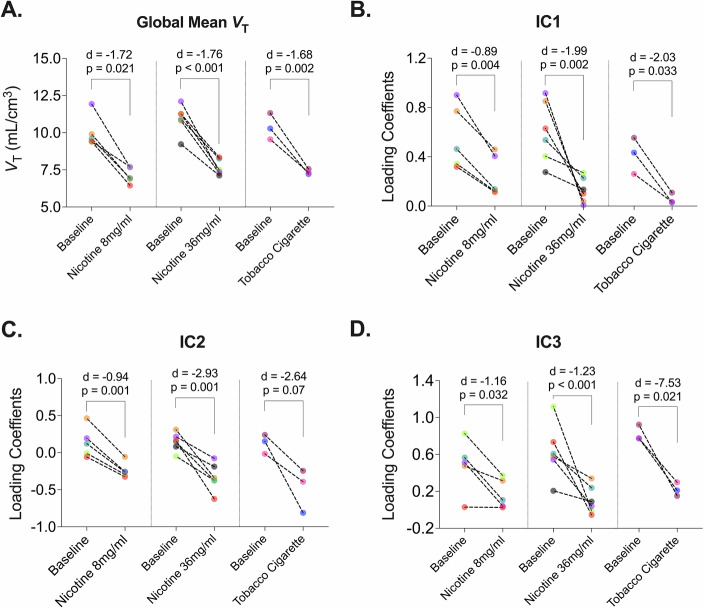
Effects of Nicotine on component loading coefficients. Comparison of global mean *V*_T_ **A** and loading coefficients for IC1 **B**, IC2 **C**, and IC3 **D** between baseline and nicotine blocking scans at two doses (8 mg/ml and 36 mg/ml) and standard cigarette from Dataset II. Cohen’s d **D** is used as the effect size measure. All *p* values are from multiple t-tests.

To validate that component loading coefficients specifically reflected β2*-nAChR specific binding rather than non-specific radiotracer uptake, loading coefficients were compared between baseline and nicotine-induced blocking scans. Given nicotine’s high affinity for nAChRs, it was expected to reduce specific binding, evident through a pronounced reduction in global mean *V*_T_ values (Fig. 2). Indeed, nicotine challenge significantly reduced loading coefficients across all conditions (Cohen’s d range: -0.89 to -7.53; detailed statistics available in Supplementary Table 2). These findings confirm that the identified independent components represent regions of specific [^18^F]Flubatine binding to nAChRs.

### Autoradiography to assess β2*-nAChR pharmacology of ICA components

To evaluate the pharmacological identity of [^18^F]Flubatine components, competition binding assays were first performed in postmortem non-human primate brains with [^18^F]Flubatine and AT1001, A85380, α-Conotoxin PIA, and α-Conotoxin MII, which have high affinity for α4β3*, α4β2*, α6β2*, and α3/α6β2* subtypes. Minimal competition was observed with AT1001 (targeting α3β4*), confirming high selectivity of [^18^F]Flubatine for β2*-nAChRs over β4*-nAChRs. In contrast, [^18^F]Flubatine was effectively displaced by A85380, α-Conotoxin PIA, and α-Conotoxin MII, with Ki values of 0.02 nM, 1.56 nM, and 4.98 nM, respectively (Supplementary Fig. 4), demonstrating specific binding of [^18^F]Flubatine at α4β2*, α6β2* and α3/α6β2*-nAChR subtypes.

**Fig. 3:**
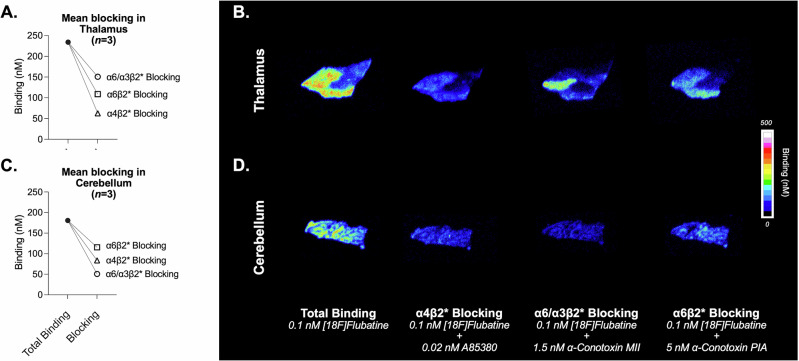
Subtype-specific blocking of [^18^F]Flubatine in thalamus and cerebellum of human post-mortem brain tissue. Mean binding (*n* = 3) is shown for total binding (0.1 nM [^18^F]Flubatine) and after blockade with A85380 (0.02 nM; α4β2*), α-conotoxin MII (1.5 nM; α6/α3β2*), and α-conotoxin PIA (5 nM; α6β2*). Connecting lines link paired measurements in thalamus **A** and the cerebellum **C**. Representative autoradiograms (left to right) display total binding and the three blocking conditions in the thalamus **B** and the cerebellum **D**. The scale denotes binding (nM).

Based on the spatial distributions of identified ICs, we hypothesized that IC1 and IC2 are associated with the α4β2* subtype, while IC3 with the α3β2* subtype. To test these associations, we conducted blocking studies using postmortem human brain tissue extracted from the thalamus and cerebellum to observe displacement patterns of [^18^F]Flubatine when co-incubated with subtype-specific ligands at their specific *K*_i_ values. In the thalamus, the displacement patterns varied across the grey matter. The highest mean thalamic blocking was observed with the α4β2* ligand (see Fig. 3A), aligning with the high prevalence and affinity of [^18^F]Flubatine for α4β2*-nAChRs, and supporting association of IC1 and IC2 with this receptor subtype. Homogeneous blocking was observed with the α4β2* ligand A85380, indicating a broad distribution of this subtype, while blocking with the α3/α6β2* and α6β2* ligands α-Conotoxin MII, and α-Conotoxin PIA, respectively, was more heterogeneous (Fig. 3B). In the cerebellum the greatest displacement of [^18^F]Flubatine binding was observed with the ligands specific for α3/α6β2* and α4β2* receptors, with less displacement by α-Conotoxin PIA (see Figs. 3C and 3D). Across three brains, the highest mean displacement occurred with the α3/α6β2* ligand -Conotoxin MII and lowest mean displacement occurred with the α6β2* ligand α-Conotoxin PIA (see Fig. 3C), supporting association of IC3 with α3β2* receptors.

### Group difference in loading coefficients between non-smokers and abstinent smokers

**Fig. 4:**
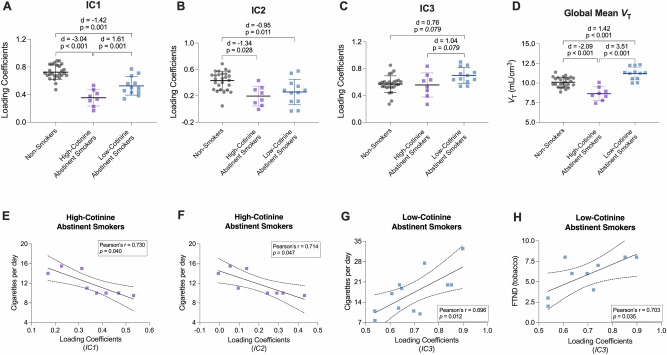
Independent Component (IC) loading coefficients in people who smoke tobacco. Top row shows group differences in IC loading coefficients for IC1 **A** IC2 **B** IC3 **C** and global mean *V*_T_ **D** between non-smokers, high-cotinine abstinent smokers and low-cotinine abstinent smokers. Cohen’s d indicate effect size, *p* values are from *post hoc* analyses with false discovery rate correction, and error bars represent standard deviations. Bottom row shows associations between loading coefficients and smoking characteristics in abstinent smokers. **E** and **F** show inverse relationships between IC1 and IC2 loading coefficients and cigarettes per day in high-cotinine abstinent smokers, respectively. **G** and **H** show positive relationships between IC3 loading coefficients and cigarettes per day and FTND tobacco scores in low-cotinine abstinent smokers. Solid lines represent the linear regression fit, dotted lines represent 95% confidence intervals, and Pearson’s r and *p*-values (uncorrected for multiple comparisons) are included.

ICA was conducted separately in non-smokers (*n* = 26) and abstinent smokers (*n* = 20) to verify consistency in component source map extraction across these cohorts. DSC comparing the groups indicated substantial overlap in extracted source maps (IC1: DSC = 0.82; IC2: DSC = 0.81; IC3: DSC = 0.83), demonstrating reliable and reproducible extraction of independent components. Therefore, the full dataset was combined for subsequent analyses comparing group differences in loading coefficients. These results are shown in Fig. 4A–D and summarized below.

Linear models were applied to assess differences in global mean *V*_T_ and loading coefficients among the three groups (non-smokers, high-cotinine abstinent smokers, and low-cotinine abstinent smokers), with age included as a covariate due to significant demographic differences across groups. Analysis of global mean *V*_T_ revealed a significant group effect (F(2,42) = 29.7, *p* < 0.001). Post hoc tests indicated that low-cotinine abstinent smokers exhibited significantly higher global mean *V*_T_ compared to non-smokers (Cohen’s d = 1.42, *p* < 0.001), whereas high-cotinine abstinent smokers showed significantly lower values relative to non-smokers (d = -2.09, *p* < 0.001), consistent with competition for nAChR binding by smoked nicotine. Additionally, low-cotinine abstinent smokers demonstrated significantly higher global mean *V*_T_ than high-cotinine abstinent smokers (d = -3.51, *p* < 0.001).

For IC1 loading coefficients, a significant group effect was observed (F(2,42) = 41.1, *p* < 0.001). Post hoc analyses demonstrated significantly lower loading coefficients in both low-cotinine (d = -1.42, *p* = 0.001) and high-cotinine (d = -3.04, *p* < 0.001) abstinent smokers compared to non-smokers. Furthermore, low-cotinine abstinent smokers showed significantly higher loading coefficients than high-cotinine abstinent smokers (d = 1.61, *p* = 0.001). Age significantly predicted IC1 loading coefficients (F(1,42) = 6.80, *p* = 0.013), with older age associated with reduced loading values, consistent with decreased availability of these nAChRs.

Analysis of IC2 loading coefficients also revealed a significant group effect (F(2,42) = 9.0, *p* < 0.001). *Post hoc* comparisons indicated that both low-cotinine (d = 0.95, *p* = 0.028) and high-cotinine abstinent smokers (d = 1.34, *p* = 0.011) exhibited significantly lower loading coefficients relative to non-smokers. No significant difference was observed between low- and high-cotinine abstinent smoker groups.

For IC3 loading coefficients, the group effect was significant (F(2,42) = 4.3, *p* = 0.020). *Post hoc* analyses suggested a trend towards higher loading coefficients in low-cotinine abstinent smokers compared to both non-smokers (d = 0.76, *p* = 0.079) and high-cotinine abstinent smokers (d = -1.04, *p* = 0.079). High-cotinine abstinent smokers did not differ significantly from non-smokers.

### Correlation between smoking characteristics and loading coefficients in abstinent smoker group

No associations between smoking-related characteristics and component loading coefficients survived FDR correction for multiple comparisons (Supplementary Table 3). However, exploratory analyses (i.e., results not corrected for multiple comparisons) revealed that in high-cotinine abstinent smokers, cigarettes per day was negatively associated with loadings in IC1 (*r* = -0.73, *p* = 0.040) and IC2 (*r* = -0.71, *p* = 0.047). In low-cotinine abstinent smokers, IC3 was positively associated with both cigarettes per day (*r* = 0.70, *p* = 0.012) and FTND tobacco scores (*r* = 0.70, *p* = 0.035). These exploratory findings are illustrated in Fig. 4E–H.

## Discussion

The use of ICA to quantify pharmacologically specific sources of radiotracer binding from a mixed-target PET radiotracer represents an approach we name **pharmaco-ICA (phICA)**. This work establishes phICA as a reproducible method to estimate a shared source of [^18^F]Flubatine specific binding corresponding to β2*-nAChR subpopulations in cerebellum. Additional components were identified in thalamus and midbrain, but pharmacological selectivity of these components could not be confirmed. The predominantly cerebellar source likely corresponds to α3β2*-nAChR specific binding as supported by pharmacological challenge studies coupled with autoradiography. The high levels of α3β2*-nAChRs in human cerebellum represent a notable species difference compared with nonhuman primate and rodent brain. ICA results indicate that the two predominantly thalamic sources (IC1 and IC2) had *lower* loading coefficients in people who smoke tobacco compared with non-smokers, and that greater nicotine use corresponds to lower component loading coefficients, suggesting that availability of the two β2*-nAChR sources may be decreased in people who smoke tobacco. In contrast, loading coefficients of IC3 (α3β2*-nAChR components) were higher in people who smoke tobacco compared with non-smokers, where higher loading corresponded to greater nicotine use and dependence severity. The results demonstrate the use of this analytic method to interrogate specific binding to β2*-nAChR.

Three reproducible components were identified by ICA. The validation of the first two components (IC1 and IC2) is challenging. These components are primarily found in the thalamus and midbrain, regions with dense α4 and β2 nAChR subunit mRNA and protein expression [38]. Nicotine challenge significantly reduced loading coefficients for both IC1 and IC2 (see Fig. 2), demonstrating that these components represent spatially coherent sources of [^18^F]Flubatine specific binding, but we cannot firmly establish further interpretation of these components. Secondary analyses of IC1 and IC2 loading coefficients revealed them to be correlated (*r*^2^ = 0.88), however, their spatial maps were distinct and highly stable across model orders, suggesting separate underlying networks with shared expression. The correlation likely reflects shared co-modulation across subjects rather than duplication. Indeed, one speculative explanation is that these components represent different ratios of α4β2 nAChR isoform expression (high-sensitivity (α4)2(β2)3 vs. low sensitivity (α4)3(β2)2 [12, 13]). Interestingly, preclinical studies show that thalamus has a higher ratio of β2:α4 subunits than cortical regions [39], consistent with greater high-sensitivity α4β2 nAChR isoforms in thalamus. Chronic nicotine does not upregulate total β2*-nAChR binding in thalamus [40, 41], while in cortex chronic nicotine increases the ratio of β2:α4 subunits and increases β2*-nAChR binding. These observations support the hypothesis that chronic nicotine can shift the stoichiometry of β2*-nAChRs from the low sensitivity to a high sensitivity isoform. The observed concurrent increase in global mean *V*_T_ with a decrease in IC1 and IC2 loadings in people who smoked is consistent with this hypothesis, however, this speculative mechanism would be challenging to confirm. Thus, we cannot make a strong claim regarding the interpretation of IC1 and IC2 beyond the fact that both represent spatially coherent sources of specific binding to β2*-nAChRs.

In contrast, IC3, which includes much of the cerebellum, could be pharmacologically validated with autoradiography studies in *postmortem* human tissue. Human autoradiography was necessary to validate this component because there are major species differences in nAChR subunit expression and PET radiotracer specific binding in cerebellum. Low, but significant, β2*-nAChR radiotracer binding is observed in rat [42, 43], but very low to negligible specific binding is observed in rhesus monkey [27, 44, 45], whereas cerebellum exhibits moderate to high nAChR radiotracer specific binding in humans, notably with particularly high specific binding for [^18^F]Flubatine [25, 32]. The blocking drugs used were α-Conotoxin MII, which has roughly equivalent affinity for α6β2 and α3β2 nAChRs, and α-Conotoxin PIA, which exhibits selectivity for α6β2 nAChR over α3β2 and α4β2 nAChRs [46, 47]. In cerebellum, α-Conotoxin MII caused the greatest reduction in total [^18^F]Flubatine binding, while α-Conotoxin PIA resulted in the smallest reduction in total [^18^F]Flubatine binding (see Fig. 3). This result supports interpretation of IC3 as representing specific binding of α3β2*-nAChRs. Indeed, substantial α3 mRNA expression has been reported in human cerebellum, particularly in the dentate and Purkinje cell layers [48, 49]. This component also includes visual pathway circuitry, previously noted to be rich in α3 (and α6) nAChR subunits [50]. Notably, IC3 $\widetilde{{V}_{{{\rm{T}}}}}$ magnitudes were a small fraction of total *V*_T_ values, corresponding well to the small fraction of α3β2*-nAChRs that contribute to total β2*-nAChR levels in these regions. Measuring α3β2*-nAChRs in living human brain could potentially provide novel insights into conditions including substance use disorders (such as nicotine [51] and alcohol [52] use), autism spectrum disorder [53], and Parkinson’s disease [54].

The presented use of phICA to separate α3β2*-nAChR specific binding from other receptor subtypes with [^18^F]Flubatine builds on previous work separating dopamine D_2_- and D_3_- related sources of [^11^C]PHNO specific binding in people with cocaine use disorder [24] as validated with D_3_-specific drug challenge [23]. Theoretically, phICA could be applied to many different mixed-target radiotracers such as [^18^F]AV-1451, which targets tau but also has affinity for monoamine oxidase B [55]; [^11^C]EKAP, which targets μ and κ opioid receptors [56], and [^11^C]LSN3172176, which targets muscarinic AChRs M1, M4 [57]. The total specific binding measured by a PET radiotracer is a sum of each receptor pool’s binding potential (*BP*_ND_), which is proportional to the ratio of target receptor density (*B*_max)_ over radiotracer affinity (*K*_D_) (${{BP}}_{{ND}}=\mathop{\sum }_{J}\frac{{B}_{\max }^{j}}{{K}_{D}^{j}}$ for *j* receptor pools). We therefore hypothesize that phICA is best suited to radiotracers with higher affinity for less densely expressed target receptor pools. Notably, this pharmacological profile is observed for both [^11^C]PHNO (D_2_R *K*_D_/*f*_ND_ ~ 11–14 nM; D_3_R *K*_D_/*f*_ND_ ~ 0.23–0.56 nM) [58] and [^18^F]Flubatine (*K*_D_ ~ 3.8, 9.2, 2.1 pM for α3β2, α4β2, α6β2, respectively) [59]. However, it is important to validate the pharmacological specificity of ICA-derived components with blocking studies, as has been done previously with imaging data [23] and as we do here with autoradiography. phICA holds promise to mitigate some of the fundamental challenges of ligand selectivity in PET radiotracer development using analytic approaches.

The current analyses add to a robust imaging literature demonstrating higher β2*-nAChR numbers in people who smoke cigarettes compared to people who never smoked cigarettes [5–7, 11, 60, 61]. Prior findings broadly report β2*-nAChR-upregulation in most brain regions. The scan-specific global mean *V*_T_ ($\bar{{x}_{j}}$ in Equation **1**), which accounts for nearly all [^18^F]Flubatine specific binding in cortex and striatum, largely replicates this result with comparable effect sizes (see Fig. 4). However, in thalamus, evidence for β2*-nAChR-upregulation from chronic nicotine is not robust [40, 62], and is reflected in mixed results from human post-mortem [63] and molecular imaging studies [6, 60]. The thalamus is technically challenging to image because β2*-nAChR radiotracers often do not achieve equilibrium conditions in this receptor rich region, although this potential source of bias was mitigated by implementing a tissue clearance correction [31]. With this safeguard, the phICA results suggest that coherent sources of β2*-nAChR binding present in thalamus (i.e., IC1, IC2) are lower during tobacco smoking abstinence compared with non-smokers, and that lower loading coefficients correspond to heavier cigarette smoking. If IC1 and IC2 indeed represent underlying pools of β2*-nAChR, this would imply that the thalamus contains populations of receptors with reduced receptor binding in combination with the upregulation of α4β2*-nAChRs captured by the global mean, resulting in a net outcome that chronic nicotine does not change total thalamic β2*-nAChR binding. While we cannot confirm the identity of IC1 and IC2 in the current work, it is interesting to note that preclinical evidence for a mechanism of nAChR downregulation has been reported for α6β2*-nAChRs lacking the β3 subunit [12, 64, 65], mainly in striatum but also in the geniculate nucleus [66]. The current autoradiography analyses were inconclusive in identifying α6β2*-nAChR among ICA components, but do indicate that α6β2*-nAChRs contribute to total [^18^F]Flubatine binding in thalamus. Nonetheless, the imaging results suggest a complex role for β2*-nAChR pools in thalamic brain regions during abstinence from chronic tobacco smoking.

The finding that IC3 loading is significantly higher during extended tobacco smoking abstinence compared to non-smokers implies an upregulation of α3β2*-nAChRs in cerebellum and optical circuitry as a result of tobacco smoking. This is consistent with preclinical reports that chronic nicotine upregulates α3β2*- nAChR [67, 68]. The α3 subunit is part of the α3/α5/β4 nAChR gene cluster that has an allelic variation that significantly increases risk of nicotine use disorder [15, 16]. While the effect of polymorphism in this gene cluster is most closely associated with the α5 subunit, this may result in part from limited tools to probe the α3 subunit (genetic KO of α3 in rodents is lethal). phICA could provide new ways to study this subunit in humans. Interestingly, α3β2*-nAChRs are less desensitized by nicotine compared with other subunits [17], suggesting that at this receptor may still be activated by endogenous acetylcholine even in the presence of chronic nicotine. Thus, it has been suggested that α3β2*-nAChRs may be a target for ameliorating cognitive deficits that can arise during attempts to quit smoking [17, 18]. Future human imaging studies could further investigate this and other roles of α3β2*-nAChR upregulation in people who smoke tobacco.

This work has several limitations. First, while careful consideration was taken to establish ICA component reproducibility within the study groups (DSC > 0.8, indicating high spatial overlap), these results should be reproduced in larger independent samples to establish generalizability. Since different PET radiotracers can have different pharmacological profiles, these components would necessarily need to be reproduced for [^18^F]Flubatine (as opposed to a different β2*-nAChR-specific radiotracer). Second, the gold standard for component validation is pharmacological blockade during in vivo imaging. Since conotoxins are toxic compounds that do not cross the blood-brain barrier well, subtype specific blocking was performed with *postmortem* autoradiography, thus limitations in translating ex vivo tissue findings to in vivo imaging results are relevant. Third, participants who were abstinent from tobacco smoking were significantly older than nonsmoking participants, however, age was included as a covariate in statistical models in an attempt to mitigate this potential confound. Fourth, groups did not have an even distribution of men and women, limiting the ability to assess previously reported sex differences in β2*-nAChR during tobacco smoking abstinence [69, 70]. Post-hoc analyses examining potential sex differences in loading coefficients found no evidence for group differences, but since this study was likely underpowered to examine potential sex differences, it remains an important consideration for future work. Finally, extension of this analytical approach to other study groups with relevant pharmacology (e.g., Parkinson’s Disease, Alzheimer’s Disease, Schizophrenia) would provide important confirmation of these findings and extend the impact of the work.

## Conclusion

In conclusion, this work applied phICA to [^18^F]Flubatine PET data, revealing three reproducible components of specific binding to β2*-nAChRs. One component, predominantly expressed in cerebellum and midbrain, likely represents α3β2*-nAChRs as supported by autoradiography in the presence of specific nAChR antagonists. The thalamic components (IC1 and IC2) were significantly lower in abstinent people who smoke compared with non-smokers, while binding to α3β2*-nAChR was significantly higher in abstinent people who smoke. Exploratory analyses provided initial evidence for greater α3β2*-nAChR binding in people who smoked greater amounts and had higher levels of nicotine dependence. These results reveal novel human evidence suggesting upregulation of α3β2*-nAChR in people who smoke tobacco, which may inform novel treatment development to help people quit smoking.

## Supplementary information

Supplementary Materials

## Data Availability

The datasets generated during and/or analyzed during the current study are available from the corresponding author upon reasonable request.

## References

[1] West R. Tobacco smoking: Health impact, prevalence, correlates and interventions. Psychol Health. 2017;32:1018–36. 28553727 10.1080/08870446.2017.1325890 PMC5490618

[2] Wittenberg RE, Wolfman SL, De Biasi M, Dani JA. Nicotinic acetylcholine receptors and nicotine addiction: a brief introduction. Neuropharmacology. 2020;177:108256. 32738308 10.1016/j.neuropharm.2020.108256 PMC7554201

[3] Picciotto MR, Zoli M, Rimondini R, Léna C, Marubio LM, Pich EM, et al. Acetylcholine receptors containing the β2 subunit are involved in the reinforcing properties of nicotine. Nature. 1998;391:173–7. 9428762 10.1038/34413

[4] Wills L, Ables JL, Braunscheidel KM, Caligiuri SPB, Elayouby KS, Fillinger C, et al. Neurobiological Mechanisms of Nicotine Reward and Aversion. Pharm Rev. 2022;74:271–310. 35017179 10.1124/pharmrev.121.000299 PMC11060337

[5] Staley JK, Krishnan-Sarin S, Cosgrove KP, Krantzler E, Frohlich E, Perry E, et al. Human tobacco smokers in early abstinence have higher levels of β2* nicotinic acetylcholine receptors than nonsmokers. J Neurosci. 2006;26:8707–14. 16928859 10.1523/JNEUROSCI.0546-06.2006 PMC6674379

[6] Mukhin AG, Kimes AS, Chefer SI, Matochik JA, Contoreggi CS, Horti AG, et al. Greater nicotinic acetylcholine receptor density in smokers than in nonsmokers: a PET study with 2-18F-FA-85380. J Nucl Med. 2008;49:1628–35. 18794265 10.2967/jnumed.108.050716 PMC2766917

[7] Mamede M, Ishizu K, Ueda M, Mukai T, Iida Y, Kawashima H, et al. Temporal change in human nicotinic acetylcholine receptor after smoking cessation: 5IA SPECT study. J Nucl Med. 2007;48:1829–35. 17942810 10.2967/jnumed.107.043471

[8] Nordberg A, Romanelli L, Sundwall A, Bianchi C, Beani L. Effect of acute and subchronic nicotine treatment on cortical acetylcholine release and on nicotinic receptors in rats and guinea-pigs. Br J Pharmacol. 1989;98:71. 2804554 10.1111/j.1476-5381.1989.tb16864.x PMC1854650

[9] Flores CM, Rogers SW, Pabreza LA, Wolfe BB, Kellar KJ. A subtype of nicotinic cholinergic receptor in rat brain is composed of alpha 4 and beta 2 subunits and is up-regulated by chronic nicotine treatment. Mol Pharmacol. 1992;41:31–37. 1732720

[10] Marks MJ, Pauly JR, Gross SD, Deneris ES, Hermans-Borgmeyer I, Heinemann SF, et al. Nicotine binding and nicotinic receptor subunit RNA after chronic nicotine treatment. J Neurosci. 1992;12:2765–84. 1613557 10.1523/JNEUROSCI.12-07-02765.1992 PMC6575859

[11] Calakos KC, Hillmer AT, Anderson JM, LeVasseur B, Baldassarri SR, Angarita GA, et al. Cholinergic system adaptations are associated with cognitive function in people recently abstinent from smoking: a (-)-[18F]flubatine PET study. Neuropsychopharmacology. 2023;48:683–9. 10.1038/s41386-023-01535-1. 36681758 10.1038/s41386-023-01535-1 PMC9938267

[12] Moroni M, Bermudez I. Stoichiometry and pharmacology of two human [alpha] 4 [beta] 2 nicotinic receptor types. J Mol Neurosci. 2006;30:95. 17192644 10.1385/JMN:30:1:95

[13] Nelson ME, Kuryatov A, Choi CH, Zhou Y, Lindstrom J. Alternate stoichiometries of α4β2 nicotinic acetylcholine receptors. Mol Pharmacol. 2003;63:332–41. 12527804 10.1124/mol.63.2.332

[14] Picciotto MR, Kenny PJ. Mechanisms of nicotine addiction. Cold Spring Harb Perspect Med. 2021;11:a039610. 32341069 10.1101/cshperspect.a039610 PMC8091956

[15] Saccone SF, Hinrichs AL, Saccone NL, Chase GA, Konvicka K, Madden PAF, et al. Cholinergic nicotinic receptor genes implicated in a nicotine dependence association study targeting 348 candidate genes with 3713 SNPs. Hum Mol Genet. 2007;16:36–49. 17135278 10.1093/hmg/ddl438 PMC2270437

[16] Berrettini W, Yuan X, Tozzi F, Song K, Francks C, Chilcoat H, et al. α-5/α-3 nicotinic receptor subunit alleles increase risk for heavy smoking. Mol Psychiatry. 2008;13:368–73. 18227835 10.1038/sj.mp.4002154 PMC2507863

[17] You S, Li X, Xiong J, Zhu X, Zhangsun D, Zhu X, et al. α-Conotoxin TxIB: a uniquely selective ligand for α6/α3β2β3 nicotinic acetylcholine receptor attenuates nicotine-induced conditioned place preference in mice. Mar Drugs. 2019;17:490. 31443523 10.3390/md17090490 PMC6780885

[18] Jackson DC, Sudweeks SN Nicotine and Alpha3beta2 Neuronal Nicotinic Acetylcholine Receptors. Neuroscience of Nicotine, Elsevier; 2019. p. 235-41.

[19] Gotti C, Guiducci S, Tedesco V, Corbioli S, Zanetti L, Moretti M, et al. Nicotinic acetylcholine receptors in the mesolimbic pathway: primary role of ventral tegmental area α6β2* receptors in mediating systemic nicotine effects on dopamine release, locomotion, and reinforcement. J Neurosci. 2010;30:5311–25. 20392953 10.1523/JNEUROSCI.5095-09.2010 PMC6632743

[20] Pang X, Liu L, Ngolab J, Zhao-Shea R, McIntosh JM, Gardner PD, et al. Habenula cholinergic neurons regulate anxiety during nicotine withdrawal via nicotinic acetylcholine receptors. Neuropharmacology. 2016;107:294–304. 27020042 10.1016/j.neuropharm.2016.03.039 PMC4982553

[21] Brunzell DH, McIntosh JM, Papke RL. Diverse strategies targeting α7 homomeric and α6β2* heteromeric nicotinic acetylcholine receptors for smoking cessation. Ann N Y Acad Sci. 2014;1327:27–45. 24730978 10.1111/nyas.12421 PMC4197117

[22] Jackson KJ, McIntosh JM, Brunzell DH, Sanjakdar SS, Damaj M. The role of α6-containing nicotinic acetylcholine receptors in nicotine reward and withdrawal. J Pharmacol Exp Ther. 2009;331:547–54. 19644040 10.1124/jpet.109.155457 PMC2775251

[23] Smart K, Gallezot J-D, Nabulsi N, Labaree D, Zheng M-Q, Huang Y, et al. Separating dopamine D2 and D3 receptor sources of [11C]-(+)-PHNO binding potential: Independent component analysis of competitive binding. Neuroimage. 2020;214:116762. 32201327 10.1016/j.neuroimage.2020.116762 PMC7263955

[24] Worhunsky PD, Matuskey D, Gallezot J-D, Gaiser EC, Nabulsi N, Angarita GA, et al. Regional and source-based patterns of [11C]-(+)-PHNO binding potential reveal concurrent alterations in dopamine D2 and D3 receptor availability in cocaine-use disorder. Neuroimage. 2017;148:343–51. 28110088 10.1016/j.neuroimage.2017.01.045 PMC5344702

[25] Baldassarri SR, Hillmer AT, Anderson JM, Jatlow P, Nabulsi N, Labaree D, et al. Use of Electronic Cigarettes Leads to Significant Beta2-Nicotinic Acetylcholine Receptor Occupancy: Evidence From a PET Imaging Study. Nicotine Tob Res. 2018;20:425–33. 28460123 10.1093/ntr/ntx091 PMC5896427

[26] Schick SF, Blount BC, Jacob PRD, Saliba NA, Bernert JT, El Hellani A, et al. Biomarkers of exposure to new and emerging tobacco delivery products. Am J Physiol Lung Cell Mol Physiol. 2017;313:L425–L452. 28522563 10.1152/ajplung.00343.2016 PMC5626373

[27] Bois F, Gallezot J-D, Zheng M-Q, Lin S-F, Esterlis I, Cosgrove KP, et al. Evaluation of [(18)F]-(-)-norchlorofluorohomoepibatidine ([(18)F]-(-)-NCFHEB) as a PET radioligand to image the nicotinic acetylcholine receptors in non-human primates. Nucl Med Biol. 2015;42:570–7. 25858513 10.1016/j.nucmedbio.2014.08.003 PMC4441617

[28] Hillmer AT, Esterlis I, Gallezot J-D, Bois F, Zheng M-Q, Nabulsi N, et al. Imaging of cerebral α4β2* nicotinic acetylcholine receptors with (−)-[18F] Flubatine PET: Implementation of bolus plus constant infusion and sensitivity to acetylcholine in human brain. Neuroimage. 2016;141:71–80. 27426839 10.1016/j.neuroimage.2016.07.026 PMC5026941

[29] Carson RE, Barker WC, Liow J-S, Johnson CA Design of a motion-compensation OSEM list-mode algorithm for resolution-recovery reconstruction for the HRRT. 2003 IEEE Nuclear Science Symposium. Conference Record, vol. 5, 2003. p. 3281-5 Vol.5.

[30] Christian BT, Vandehey NT, Floberg JM, Mistretta CA. Dynamic PET denoising with HYPR processing. J Nucl Med. 2010;51:1147–54. 20554743 10.2967/jnumed.109.073999 PMC3250311

[31] Hillmer AT, Carson RE. Quantification of PET infusion studies without true equilibrium: A tissue clearance correction. J Cereb Blood Flow Metab. 2020;40:860–74. 31088233 10.1177/0271678X19850000 PMC7168787

[32] Bhatt S, Hillmer AT, Nabulsi N, Matuskey D, Lim K, Lin S-F, et al. Evaluation of (-)-[18F]Flubatine-specific binding: Implications for reference region approaches. Synapse. 2018;72:e22016. 10.1002/syn.22016 PMC6547815 29105121

[33] Bell AJ, Sejnowski TJ. An information-maximization approach to blind separation and blind deconvolution. Neural Comput. 1995;7:1129–59. 7584893 10.1162/neco.1995.7.6.1129

[34] Himberg J, Hyvärinen A, Esposito F. Validating the independent components of neuroimaging time series via clustering and visualization. Neuroimage. 2004;22:1214–22. 15219593 10.1016/j.neuroimage.2004.03.027

[35] Reuter M, Schmansky NJ, Rosas HD, Fischl B. Within-subject template estimation for unbiased longitudinal image analysis. Neuroimage. 2012;61:1402–18. 22430496 10.1016/j.neuroimage.2012.02.084 PMC3389460

[36] Iglesias JE, Insausti R, Lerma-Usabiaga G, Bocchetta M, Van Leemput K, Greve DN, et al. A probabilistic atlas of the human thalamic nuclei combining ex vivo MRI and histology. Neuroimage. 2018;183:314–26. 30121337 10.1016/j.neuroimage.2018.08.012 PMC6215335

[37] Xiao Y, Fonov V, Bériault S, Subaie FA, Chakravarty MM, Sadikot AF, et al. Multi-contrast unbiased MRI atlas of a Parkinson’s disease population. Int J Comput Assist Radiol Surg. 2015;10:329–41. 24841147 10.1007/s11548-014-1068-y

[38] Hawrylycz MJ, Lein ES, Guillozet-Bongaarts AL, Shen EH, Ng L, Miller JA, et al. An anatomically comprehensive atlas of the adult human brain transcriptome. Nature. 2012;489:391–9. 22996553 10.1038/nature11405 PMC4243026

[39] Gotti C, Moretti M, Meinerz NM, Clementi F, Gaimarri A, Collins AC, et al. Partial Deletion of the Nicotinic Cholinergic Receptor α4 or β2 Subunit Genes Changes the Acetylcholine Sensitivity of Receptor-Mediated 86Rb+ Efflux in Cortex and Thalamus and Alters Relative Expression of α4 and β2 Subunits. Mol Pharm. 2008;73:1796–807. 10.1124/mol.108.045203 18337473

[40] Fasoli F, Moretti M, Zoli M, Pistillo F, Crespi A, Clementi F, et al. In vivo chronic nicotine exposure differentially and reversibly affects upregulation and stoichiometry of α4β2 nicotinic receptors in cortex and thalamus. Neuropharmacology. 2016;108:324–31. 27157710 10.1016/j.neuropharm.2016.04.048

[41] Fu X, Moonschi FH, Fox-Loe AM, Snell AA, Hopkins DM, Avelar AJ, et al. Brain Region Specific Single-Molecule Fluorescence Imaging. Anal Chem. 2019;91:10125–31. 31298524 10.1021/acs.analchem.9b02133 PMC8542424

[42] Vaupel DB, Stein EA, Mukhin AG. Quantification of α4β2* nicotinic receptors in the rat brain with microPET® and 2-[18F] FA-85380. Neuroimage. 2007;34:1352–62. 17187994 10.1016/j.neuroimage.2006.10.036 PMC2023973

[43] Hillmer AT, Wooten DW, Farhoud M, Barnhart TE, Mukherjee J, Christian BT. The effects of lobeline on α4β2* nicotinic acetylcholine receptor binding and uptake of [18F]nifene in rats. J Neurosci Methods. 2013;214:163–9. 23370310 10.1016/j.jneumeth.2013.01.018 PMC3644313

[44] Hillmer AT, Wooten DW, Slesarev MS, Ahlers EO, Barnhart TE, Murali D, et al. PET imaging of α4β2* nicotinic acetylcholine receptors: quantitative analysis of 18F-nifene kinetics in the nonhuman primate. J Nucl Med. 2012;53:1471–80. 22851633 10.2967/jnumed.112.103846 PMC3580212

[45] Chefer SI, London ED, Koren AO, Pavlova OA, Kurian V, Kimes AS, et al. Graphical analysis of 2-[18F] FA binding to nicotinic acetylcholine receptors in rhesus monkey brain. Synapse. 2003;48:25–34. 12557269 10.1002/syn.10180

[46] Dowell C, Olivera BM, Garrett JE, Staheli ST, Watkins M, Kuryatov A, et al. α-Conotoxin PIA Is Selective for α6 Subunit-Containing Nicotinic Acetylcholine Receptors. J Neurosci. 2003;23:8445–52. 13679412 10.1523/JNEUROSCI.23-24-08445.2003 PMC6740366

[47] Cuny H, Yu R, Tae H-S, Kompella SN, Adams DJ. α-Conotoxins active at α3-containing nicotinic acetylcholine receptors and their molecular determinants for selective inhibition. Br J Pharmacol. 2018;175:1855–68. 28477355 10.1111/bph.13852 PMC5979624

[48] Graham A, Court JA, Martin-Ruiz CM, Jaros E, Perry R, Volsen SG, et al. Immunohistochemical localisation of nicotinic acetylcholine receptor subunits in human cerebellum. Neuroscience. 2002;113:493–507. 12150770 10.1016/s0306-4522(02)00223-3

[49] Hellström-Lindahl E, Mousavi M, Zhang X, Ravid R, Nordberg A. Regional distribution of nicotinic receptor subunit mRNAs in human brain: comparison between Alzheimer and normal brain. Mol Brain Res. 1999;66:94–103. 10095081 10.1016/s0169-328x(99)00030-3

[50] Gotti C, Moretti M, Zanardi A, Gaimarri A, Champtiaux N, Changeux J-P, et al. Heterogeneity and Selective Targeting of Neuronal Nicotinic Acetylcholine Receptor (nAChR) Subtypes Expressed on Retinal Afferents of the Superior Colliculus and Lateral Geniculate Nucleus: Identification of a New Native nAChR Subtype α3β2(α5 or β3) Enriched in Retinocollicular Afferents. Mol Pharm. 2005;68:1162–71. 10.1124/mol.105.015925 16049166

[51] Dani JA Chapter One - Neuronal Nicotinic Acetylcholine Receptor Structure and Function and Response to Nicotine. In: De Biasi M, editor. International Review of Neurobiology, vol. 124, Academic Press; 2015. p. 3–19. 10.1016/bs.irn.2015.07.001 PMC4795468 26472524

[52] Moulton EA, Elman I, Becerra LR, Goldstein RZ, Borsook D. The cerebellum and addiction: insights gained from neuroimaging research. Add Biol. 2014;19:317–31. 10.1111/adb.12101 PMC4031616 24851284

[53] Lee M, Martin-Ruiz C, Graham A, Court J, Jaros E, Perry R, et al. Nicotinic receptor abnormalities in the cerebellar cortex in autism. Brain. 2002;125:1483–95. 12076999 10.1093/brain/awf160

[54] Bohnen NI, Yarnall AJ, Weil RS, Moro E, Moehle MS, Borghammer P, et al. Cholinergic system changes in Parkinson’s disease: emerging therapeutic approaches. Lancet Neurol. 2022;21:381–92. 35131038 10.1016/S1474-4422(21)00377-X PMC8985079

[55] Murugan NA, Chiotis K, Rodriguez-Vieitez E, Lemoine L, Ågren H, Nordberg A. Cross-interaction of tau PET tracers with monoamine oxidase B: evidence from in silico modelling and in vivo imaging. Eur J Nucl Med Mol Imaging. 2019;46:1369–82. 30919054 10.1007/s00259-019-04305-8 PMC6486902

[56] Naganawa M, Li S, Nabulsi N, Lin S-F, Labaree D, Ropchan J, et al. Kinetic Modeling and Test–Retest Reproducibility of 11C-EKAP and 11C-FEKAP, Novel Agonist Radiotracers for PET Imaging of the κ-Opioid Receptor in Humans. J Nucl Med. 2020;61:1636–42. 32169917 10.2967/jnumed.119.227694 PMC9364890

[57] Naganawa M, Nabulsi N, Henry S, Matuskey D, Lin S-F, Slieker L, et al. First-in-human assessment of 11C-LSN3172176, an M1 muscarinic acetylcholine receptor PET radiotracer. J Nucl Med. 2021;62:553–60. 32859711 10.2967/jnumed.120.246967 PMC8049371

[58] Gallezot J-D, Beaver JD, Gunn RN, Nabulsi N, Weinzimmer D, Singhal T, et al. Affinity and selectivity of [11C]-(+)-PHNO for the D3 and D2 receptors in the rhesus monkey brain in vivo. Synapse. 2012;66:489–500. 22213512 10.1002/syn.21535

[59] Avalos M, Parker MJ, Maddox FN, Carroll FI, Luetje CW. Effects of pyridine ring substitutions on affinity, efficacy, and subtype selectivity of neuronal nicotinic receptor agonist epibatidine. J Pharm Exp Ther. 2002;302:1246–52. 10.1124/jpet.102.035899 12183686

[60] Cosgrove KP, Batis J, Bois F, Maciejewski PK, Esterlis I, Kloczynski T, et al. β2-nicotinic acetylcholine receptor availability during acute and prolonged abstinence from tobacco smoking. Arch Gen Psychiatry. 2009;66:666–76. 19487632 10.1001/archgenpsychiatry.2009.41 PMC2796827

[61] Wüllner U, Gündisch D, Herzog H, Minnerop M, Joe A, Warnecke M, et al. Smoking upregulates α4β2* nicotinic acetylcholine receptors in the human brain. Neurosci Lett. 2008;430:34–37. 17997038 10.1016/j.neulet.2007.10.011

[62] Henderson BJ, Lester HA. Inside-out neuropharmacology of nicotinic drugs. Neuropharmacology. 2015;96:178–93. 25660637 10.1016/j.neuropharm.2015.01.022 PMC4486611

[63] Breese CR, Marks MJ, Logel J, Adams CE, Sullivan B, Collins AC, et al. Effect of Smoking History on [3H]Nicotine Binding in Human Postmortem Brain1. The. J Pharmacol Exp Ther. 1997;282:7–13. 9223534

[64] Moretti M, Mugnaini M, Tessari M, Zoli M, Gaimarri A, Manfredi I, et al. A Comparative Study of the Effects of the Intravenous Self-Administration or Subcutaneous Minipump Infusion of Nicotine on the Expression of Brain Neuronal Nicotinic Receptor Subtypes. Mol Pharmacol. 2010;78:287–96. 20439469 10.1124/mol.110.064071

[65] Perry DC, Mao D, Gold AB, McIntosh JM, Pezzullo JC, Kellar KJ. Chronic Nicotine Differentially Regulates α6- and β3-Containing Nicotinic Cholinergic Receptors in Rat Brain. The. J Pharmacol Exp Ther. 2007;322:306–15. 17446303 10.1124/jpet.107.121228

[66] Marks MJ, Grady SR, Salminen O, Paley MA, Wageman CR, McIntosh JM, et al. α6β2*-subtype nicotinic acetylcholine receptors are more sensitive than α4β2*-subtype receptors to regulation by chronic nicotine administration. J Neurochem. 2014;130:185–98. 24661093 10.1111/jnc.12721 PMC4107044

[67] Ke L, Eisenhour CM, Bencherif M, Lukas RJ. Effects of chronic nicotine treatment on expression of diverse nicotinic acetylcholine receptor subtypes. I. Dose-and time-dependent effects of nicotine treatment. The. J Pharmacol Exp Ther. 1998;286:825–40. 9694939

[68] Olale F, Gerzanich V, Kuryatov A, Wang F, Lindstrom J. Chronic nicotine exposure differentially affects the function of human α3, α4, and α7 neuronal nicotinic receptor subtypes. J Pharmacol Exp Ther. 1997;283:675–83. 9353385

[69] Cosgrove KP, Esterlis I, McKee SA, Bois F, Seibyl JP, Mazure CM, et al. Sex differences in availability of β2*-nicotinic acetylcholine receptors in recently abstinent tobacco smokers. Arch Gen Psychiatry. 2012;69:418–27. 22474108 10.1001/archgenpsychiatry.2011.1465 PMC3508698

[70] Moen JK, Lee AM. Sex differences in the nicotinic acetylcholine receptor system of rodents: Impacts on nicotine and alcohol reward behaviors. Front Neurosci. 2021;15:745783. 34621155 10.3389/fnins.2021.745783 PMC8490611

