# Supplementary material for: Multiple sources of β2*-nicotinic acetylcholine receptor binding are differentially affected during tobacco smoking abstinence as revealed by Independent Component Analysis of [18F]Flubatine PET images
Source: Neuropsychopharmacology. 2026 Jan 20;51(7):1207–16. doi: 10.1038/s41386-025-02311-z (PMC13213061; doi:10.1038/s41386-025-02311-z)
Supplement: Supplementary file 1 — Supplementary Materials [file 41386_2025_2311_MOESM1_ESM.docx]

# **Supplementary Information**

# Multiple sources of β2*-nicotinic acetylcholine receptor subtypes are differentially affected during tobacco smoking abstinence as revealed by Independent Component Analysis of [^18^F]Flubatine PET images.

Nakul R. Raval^1,2,3^, Katina C. Calakos^1^, Mike Wenn^1,2^, Anita Huttner^4^**,** Irina Esterlis^1,3^, Henry Huang^1,2^, Vince D. Calhoun^5^, Marina R. Picciotto^3^, Kelly P. Cosgrove^2,3^, Ansel T. Hillmer^1,2,3^

1. Department of Radiology and Biomedical Imaging, Yale University School of Medicine, New Haven, CT, USA
2. Yale PET Center, Department of Radiology and Biomedical Imaging, Yale University School of Medicine, New Haven, CT, USA
3. Department of Psychiatry, Yale University School of Medicine, New Haven, CT, USA
4. Department of Pathology, Yale University School of Medicine, New Haven, CT, USA
5. Tri-institutional Center for Translational Research in Neuroimaging and Data Science (TReNDS), Georgia State, Georgia Tech, Emory, Atlanta, GA
6. Department of Radiology, University of Michigan, Ann Arbor, MI, USA

**Contents:**

**Supplementary Figure 1:** Mask for Independent Component Analysis

**Supplementary Figure 2:** Thalamic ICA Spatial Components and Anatomical Parcellation

**Supplementary Methods:** [^18^F]Flubatine Autoradiography in Nonhuman Primate Brain

**Supplementary Figure 3**: Results of the [^18^F]Flubatine Saturation Binding Assay

**Supplementary Figure 4**: Results of the [^18^F]Flubatine Competition Binding Assay

**Supplementary Table 1:** Demography and Neuropathological Characterization of PostMortem Human Brain Samples used for Autoradiography

**Supplementary Table 2:** Pairwise Comparisons Between Baseline and Challenge Conditions

**Supplementary Table 3:** Correlation between Smoking Characteristics and Loading Coefficients in Abstinent Smokers

## ***Mask for Independent Component Analysis***


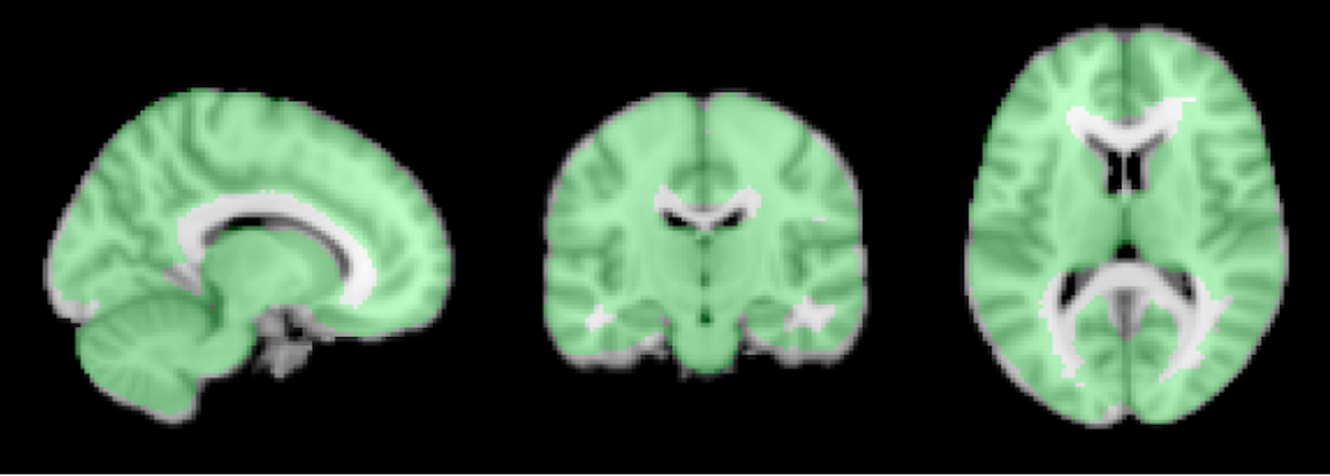

**Supplementary Figure 1.** Conjunction brain mask (green) applied to restrict the analysis to voxels exhibiting a mean distribution volume (*V*_T_) greater than 8 across subjects. The mask is overlaid on the MNI152 T1-weighted template for anatomical reference, ensuring that analyses are confined to relevant regions of interest with sufficient signal.

## ***Thalamic ICA Spatial Components and Anatomical Parcellation***

**
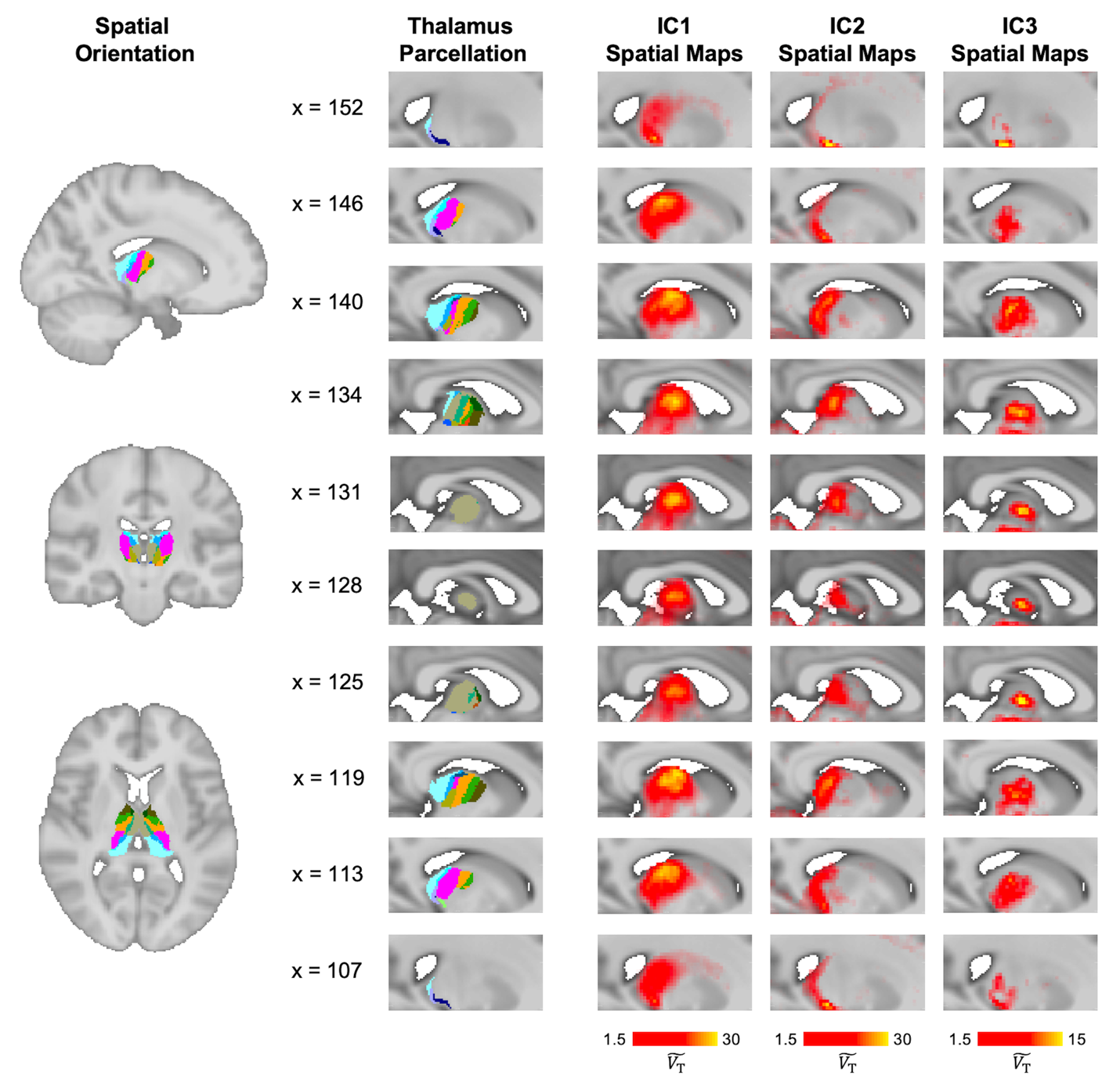
**

**Supplementary Figure 2.** Spatial maps of independent components (IC1, IC2, and IC3) zoomed in on the thalamus derived from independent component analysis (ICA) of [^18^F]Flubatine PET baseline scans in non-smokers (*n*=26). The images are displayed in the sagittal plane (x-axis: lateral to medial and back to lateral). Heatmaps in the IC columns represent $\tilde{V_{T}}$ values within each component. The first column shows the thalamic parcellation overlaid on the anatomical reference. Thalamic regions are color-coded according to the standard FreeSurfer lookup table, as visualized in FreeView. These default colors will automatically appear when the same regions of interest (ROIs) are opened in FreeView. The Anteroventral nucleus is shown in green (RGB: 0, 85, 0), the Central Medial nucleus in orange (170, 85, 0), the Central Lateral nucleus in dark green (0, 170, 0), the Central Medial nucleus in olive (170, 170, 0), the Lateral Dorsal nucleus in yellow-green (170, 255, 0), the Lateral Geniculate Nucleus in dark blue (0, 0, 127), the Lateral Posterior nucleus in blue-teal (0, 85, 127), the Lateral Suprageniculate nucleus in light purple (170, 85, 127), the Mediodorsal lateral part in sea green (0, 170, 127), the Mediodorsal medial part in tan (170, 170, 127), the Medial Geniculate Nucleus in light olive (170, 255, 127), the Medial Ventral nucleus (Reuniens) in bright blue (0, 0, 255), the Paracentral nucleus in purple (170, 0, 255), the Parafascicular nucleus in deep blue (0, 85, 255), the Paratenial nucleus in violet (170, 85, 255), the Anterior Pulvinar in cyan (0, 170, 255), the Inferior Pulvinar in light blue (170, 170, 255), the Lateral Pulvinar in aqua (0, 255, 255), the Medial Pulvinar in pale cyan (170, 255, 255), the Reticular nucleus in red (255, 0, 0), the Ventral Anterior nucleus in olive brown (85, 85, 0), the Ventral Anterior magnocellular part in orange-red (255, 85, 0), the Ventral Lateral anterior part in yellow-green (85, 170, 0), the Ventral Lateral posterior part in orange-yellow (255, 170, 0), the Ventral Medial nucleus in light green (85, 255, 0), the Ventral Posterolateral nucleus in magenta (255, 0, 255), the Paraventricular nucleus in deep purple (120, 18, 134), the Medial Medial Pulvinar in pale cyan (170, 255, 255), and the Lateral Medial Pulvinar in sky blue (140, 240, 255).

## **[^18^F]Flubatine Autoradiography in Non-human Primate Brains**

**Post-Mortem Brain Tissue Preparation:** Frozen brain sections from around the thalamus were obtained from three non-human primates (NHPs) used as controls in a previous study [66]. Brains were stored at −80°C. The tissue was cryosectioned into 20 µm thick slices using a cryostat (Leica CM1800, Leica Biosystems, Buffalo Grove, IL, USA). The sections were then mounted onto Superfrost Plus™ adhesion microscope slides (Thermo Fisher Scientific, Waltham, MA, USA) and stored at −20°C until use.

**Saturation Binding Assays to Determine [^18^F]Flubatine *K*_D_:** To determine the equilibrium dissociation constant (*K*_D_) of [^18^F]Flubatine, a saturation binding assay was conducted. The cryosections were thawed to room temperature for approximately 45 minutes before prewashing twice for 10 minutes each in assay buffer (50 mM Tris-HCl containing 0.5% bovine serum albumin (BSA), pH 7.4). The sections were then incubated for 60 minutes in assay buffer with varying concentrations of [^18^F]Flubatine (0.1 to 12 nM) to measure total binding (TB). Non-specific binding (NSB) was measured in adjacent sections using the same concentrations of [^18^F]Flubatine in the presence of 10 µM nicotine. After incubation, the sections were washed three times for 5 minutes each in cold washing buffer (50 mM Tris-HCl, pH 7.4), followed by a rapid rinse in 4°C deionized water. After air-drying, the slides were subjected to autoradiography using phosphor image plates (BAS-MS2025, Science Imaging Scandinavia AB, Nacka, Sweden), with exposure times ranging from 45 to 60 minutes. The plates were then scanned using an Amersham™ Typhoon™ IP (Cytiva, Uppsala, Sweden) at a resolution of 10 µm. Calibration, quantification, and data analysis were performed using ImageJ software (NIH, Bethesda, MD, USA).

**Competition Binding Assay To Determine [^18^F]Flubatine *K*_i_ Against Selective Ligands:** Following the saturation binding assay, a competition binding assay was performed at the *K*_D_ concentration of [^18^F]Flubatine (0.1 nM) to evaluate the selectivity of [^18^F]Flubatine for β2*-nAChRs over other subtypes. Sections were incubated with 0.1 nM [^18^F]Flubatine in the presence of varying concentrations of subtype-specific ligands, including A85380 (α4β2*), α-Conotoxin PIA (α6β2*), and α-Conotoxin MII (α3/α6β2*). Additionally, AT1001 (α3β4*) was tested in NHP brain sections to demonstrate the selectivity of [^18^F]Flubatine for β2*-nAChRs over β3*-nAChRs. The same washing, drying, and autoradiography procedures as described in the saturation binding assay were followed. The specific binding (%Binding) was calculated as follows:

$$\%Binding = \left( \frac{Specific Binding (SB)}{Total Binding (TB)} \right) \times100$$

where Specific Binding (SB) is the difference between TB and NSB. The *K*_i_ values were determined from competition curves using nonlinear regression in GraphPad Prism.

**Results of the [^18^F]Flubatine Saturation Binding Assay:** The saturation binding assay determined that [^18^F]Flubatine binds with high affinity to nAChRs, yielding a *K*_D_ value of 0.13 nM (95% CI: 0.09-0.18 nM) (**Supplementary Figure 3**).


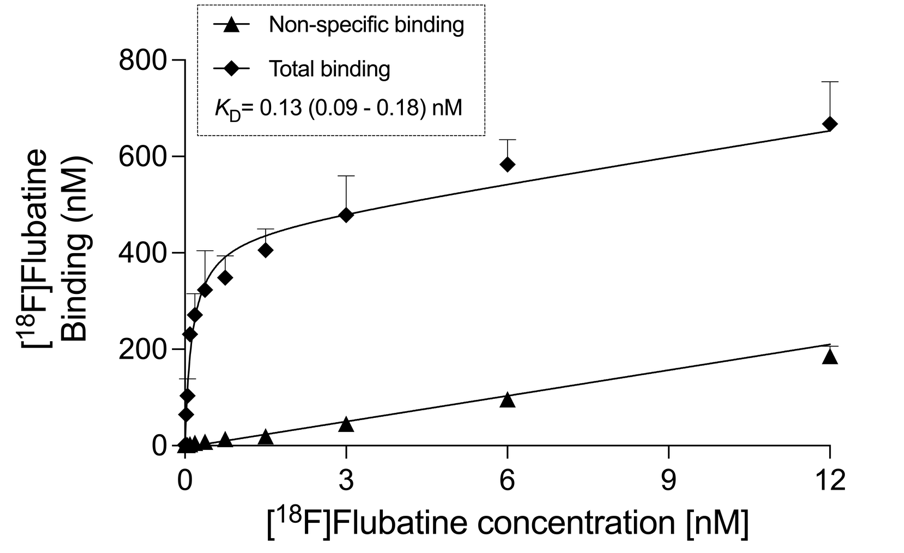


**Supplementary Figure 3.** Saturation binding assay of [^18^F]Flubatine in thalamic brain tissue from non-human primates (*n*=3). *K*_D_ was calculated using non-specific binding (NSB) determined from sections incubated with 10 µM nicotine. The *K*_D_ (inserted) value was estimated to be 0.17 nM (95% CI: 0.11 - 0.28 nM).

**Results of the [^18^F]Flubatine Competition Binding Assay:** Competition binding assays revealed that [^18^F]Flubatine binding effectively displaced by A85380, α-Conotoxin PIA, α-Conotoxin MII, with *K*_i_ values of 0.02 nM, 1.56 nM, and 4.98 nM, respectively (**Supplementary Figure 4**). The results indicate that [^18^F]Flubatine has a strong preference for α4β2*, α6β2* and α3/α6β2* subtypes. The ligand AT1001, targeting α3β4*, showed minimal competition, confirming the high selectivity of [^18^F]Flubatine for β2*-nAChRs (**Supplementary Figure 4**).


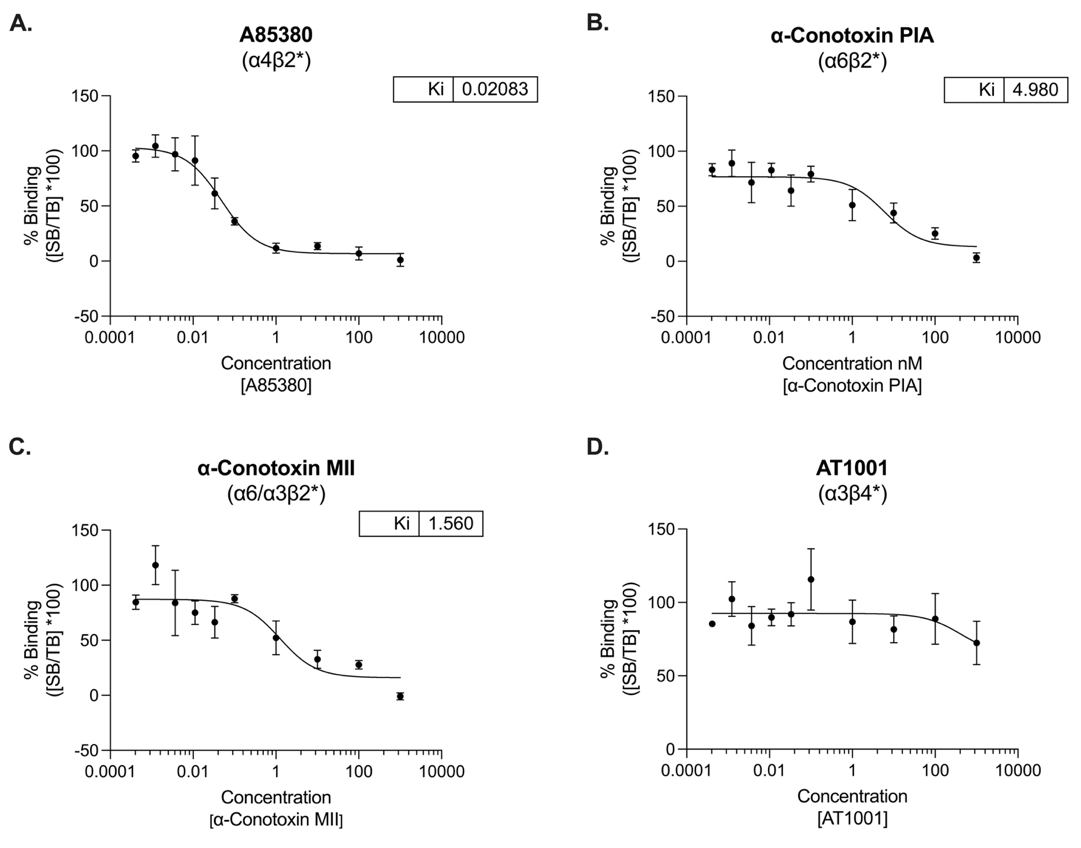


**Supplementary Figure 4.** Competition binding assays in thalamic brain sections from non-human primates (*n*=3) using [^18^F]Flubatine (0.1 nM) in the presence of varying concentrations of β2*-nAChR subtype-specific ligands. A) A85380 (α4β2*) showed strong competition with [18F]Flubatine, yielding a Ki of 0.02083 nM. B) α-Conotoxin PIA (α6β2*) competed with a *K*_i_ of 4.98 nM. C) α-Conotoxin MII (α3/α6β2*) competed with a *K*_i_ of 1.56 nM. D) AT1001 (α3β4*) demonstrated minimal competition, indicating the high selectivity of [^18^F]Flubatine for β2*-nAChRs.

**Supplementary Table 1.** Demographic and neuropathological characteristics of postmortem human brain samples used for autoradiography. PMI: postmortem interval; NIA: National Institute on Aging; A: amyloid pathology; B: Braak stage for neurofibrillary tangles; C: CERAD neuritic plaque score.

| **Supplementary Table 1: Postmortem Human brain Samples** | | | | |
| --- | --- | --- | --- | --- |
| **Postmortem human brain** | **Patient age** | **Sex** | **PMI**  **(hours)** | **NIA classification** |
| Brain 1 | 92 | Male | 22 | A1 B1 C0 |
| Brain 2 | 81 | Male | 34 | A1 B1 C0 |
| Brain 3 | 88 | Female | 6 | A0 B1 C0 |

**Supplementary Table 2:** Pairwise comparisons between baseline and nicotine/tobacco challenge conditions were performed to assess changes in global mean *V*_T_ and component loading coefficients (IC1, IC2, IC3). Cohen’s d values indicate the magnitude of within-subject effects. P-values were calculated using paired comparisons and corrected for multiple comparisons using the false discovery rate (FDR) method. Both unadjusted and FDR-adjusted p-values are reported.

| **Supplementary Table 2: Nicotine Blocking on Global Mean *V*_T_ and Component Loadings** | | | | |
| --- | --- | --- | --- | --- |
| **Measure** | **Comparison** | **Cohen's d** | **p value** | **FDR-adjusted p value** |
| Global Mean *V*_T_ | Baseline vs. Nicotine 8 mg/ml | -1.72 | 0.021 | 0.028 |
| Global Mean *V*_T_ | Baseline vs. Nicotine 36 mg/ml | -1.76 | < 0.001 | 0.002 |
| Global Mean *V*_T_ | Baseline vs. Tobacco Cigarette | -1.68 | 0.0002 | 0.002 |
| IC1 | Baseline vs. Nicotine 8 mg/ml | -0.89 | 0.004 | 0.007 |
| IC1 | Baseline vs. Nicotine 36 mg/ml | -1.99 | 0.002 | 0.004 |
| IC1 | Baseline vs. Tobacco Cigarette | -2.03 | 0.033 | 0.036 |
| IC2 | Baseline vs. Nicotine 8 mg/ml | -0.94 | 0.001 | 0.002 |
| IC2 | Baseline vs. Nicotine 36 mg/ml | -2.93 | 0.001 | 0.002 |
| IC2 | Baseline vs. Tobacco Cigarette | -2.64 | 0.07 | 0.07 |
| IC3 | Baseline vs. Nicotine 8 mg/ml | -1.16 | 0.032 | 0.036 |
| IC3 | Baseline vs. Nicotine 36 mg/ml | -1.23 | < 0.001 | 0.002 |
| IC3 | Baseline vs. Tobacco Cigarette | -7.53 | 0.021 | 0.028 |

**Supplementary Table 3:** Pearson correlation coefficients (r), uncorrected p-values, and FDR-adjusted p-values for associations between PET loading coefficients (IC1, IC2, IC3) and smoking characteristics in high-cotinine and low-cotinine abstinent smokers. None of the correlations survived FDR correction for multiple comparisons. ​

| **Supplementary Table 3: Correlation between Smoking Characteristics and Loading Coefficients in between Abstinent Smokers** | | | | | |
| --- | --- | --- | --- | --- | --- |
| **Group** | **Loading Coefficient** | **Characteristic** | **Pearson’s r** | **p value** | **FDR-adjusted p value** |
| High-Cotinine Abstinent Smokers | IC1 | Cigarettes/day | -0.7305 | 0.0396* | 0.2097 |
|  | IC2 | Cigarettes/day | -0.7141 | 0.0466* | 0.2097 |
|  | IC3 | Cigarettes/day | -0.3348 | 0.4176 | 0.7052 |
|  | IC1 | Years of smoking | 0.2512 | 0.5485 | 0.7052 |
|  | IC2 | Years of smoking | 0.267 | 0.5227 | 0.7052 |
|  | IC3 | Years of smoking | -0.2869 | 0.4909 | 0.7052 |
|  | IC1 | FTND (tobacco) | -0.1301 | 0.7588 | 0.7588 |
|  | IC2 | FTND (tobacco) | -0.1632 | 0.6994 | 0.7588 |
|  | IC3 | FTND (tobacco) | -0.6577 | 0.0763 | 0.2288 |
|  | | | | | |
| Low-Cotinine Abstinent Smokers | IC1 | Cigarettes/day | -0.2194 | 0.4932 | 0.6341 |
|  | IC2 | Cigarettes/day | -0.261 | 0.4126 | 0.6341 |
|  | IC3 | Cigarettes/day | 0.6957 | 0.012* | 0.108 |
|  | IC1 | Years of smoking | -0.3267 | 0.2999 | 0.6341 |
|  | IC2 | Years of smoking | -0.253 | 0.4275 | 0.6341 |
|  | IC3 | Years of smoking | 0.18 | 0.5755 | 0.6475 |
|  | IC1 | FTND (tobacco) | -0.0153 | 0.9689 | 0.9689 |
|  | IC2 | FTND (tobacco) | -0.3297 | 0.3863 | 0.6341 |
|  | IC3 | FTND (tobacco) | 0.7028 | 0.0347* | 0.1563 |
